# Unusual presentation of glioblastoma in the brainstem: a case report of a diffuse pontine glioblastoma multiforme and surgical management

**DOI:** 10.3389/fonc.2024.1279897

**Published:** 2024-03-13

**Authors:** Brandon Edelbach, Vadim Gospodarev, Miguel Lopez-Gonzalez, Jeremy Deisch, Maninder Kaur

**Affiliations:** ^1^ School of Medicine, Loma Linda University, Loma Linda, CA, United States; ^2^ Department of Neurosurgery, Loma Linda University Medical Center, Loma Linda, CA, United States; ^3^ Department of Pathology, Loma Linda University Medical Center, Loma Linda, CA, United States

**Keywords:** glioblastoma multiforme, pons, radiation therapy, temozolomide therapy, infratentorial glioblastoma multiforme, molecular subtyping, STUPP regimen

## Abstract

Diffuse pontine glioblastoma multiforme is a rare subtype of glioblastoma associated with a poor prognosis. In this case report, we present a unique case of diffuse primary pontine glioblastoma multiforme in a patient without any supratentorial lesions. We review the symptoms, treatment options, and case management of patients with infratentorial glioblastoma multiforme and compare these with our patient. Our patient presented with symptoms including progressive diplopia, gait disturbance, and lower extremity weakness. Magnetic resonance imaging revealed a diffuse lesion involving the pons and biopsy revealed only mildly-atypical glial infiltrates. Consequentially, diagnosis was driven by genetic analysis. Due to the location of the tumor, surgery was not considered a viable option. Instead, the patient received radiation therapy along with concomitant and adjuvant temozolomide chemotherapy which has resulted in improvement of symptoms. This case highlights the challenges of managing diffuse primary pontine glioblastoma multiforme and the need for more effective treatment options for this rare subtype of glioblastoma. Despite aggressive treatment, the prognosis for patients with infratentorial glioblastoma multiforme remains poor, with a median survival time of less than a year. Further research is needed to improve our understanding of the biology and optimal management of this disease.

## 1 Introduction

One of the most common malignancies of the central nervous system (CNS) are glioblastomas representing 14.3% of all CNS tumors and 49.1% of all malignant CNS tumors. Glioblastoma multiforme (GBM) are WHO grade IV tumors which are characteristically highly vascularized and possess a significant propensity to disperse throughout the brain parenchyma (1). These tumors are most commonly located in the frontal lobe of the supratentorial compartment (2). GBM has an increased incidence among men ages 70-85 years; furthermore, these tumors are approximately two times more prevalent among Caucasians than African-American patients (3). GBM had one of the lowest survival rate of 8 months with only 6.8% of patients surviving beyond 5 years (3). Additionally, older patients diagnosed with GBM often have worse prognosis (4). GBM often presents non-specifically with headaches, seizures, paresthesia, vision changes and personality changes.

The genetic morphology of GBM is crucial from not only a therapeutic perspective but also prognostically. GBMs appear to be derived from neural progenitors (5). These tumors arise from either neural stem cells or differentiated astrocytes, however there is still some debate over the origin of GBMs (6). Several common GBM biomarkers include O-6-methylguanine DNA methyltransferase (MGMT) and DNA wide methylation (marked by CpG island methylation) which were both associated better prognosis, while amplification of the epidermal growth factor receptor (EGFR) and the IDH wild type which is associated with telomerase reverse transcriptase (TERT) promoter methylation were both associated with decreased survival times (3). Mutations of the TERT gene occurred at a high frequency (seen in 70% of glioblastomas) (7, 8). Additionally the EGFR mutations were present in 57% of patients (9).

In order to improve diagnosis and treatment of these genetically heterogeneous tumors, a subclassification scheme was proposed by Philips et al. which divided GBMs into proneural, proliferative, and mesenchymal subclasses. Tumor subdivision was based on biomarker expression. The Mesenchymal and proliferative subclasses had an astrocytic morphology, often presenting in patients over 50 years old, and were associated with Akt activation, PTEN loss and gain of chromosome 7 and loss of chromosome 10. The mesenchymal subclass was characterized by the VEGF and CD44 markers while the proliferative subclass was associated with PCNA and TOP2A markers. The proneural subclass was found to develop in significantly younger patients, averaging 40 years old, and was associated with the longest survival time of the three subclasses and was associated with markers DLL3, BCAN, and Olig2 and was found to have PTEN intact, EGFR normal and Notch activation (10). These subclasses were proposed to exist along a spectrum with proneural and proliferative subclasses progressing to mesenchymal subclass (11). The prognostic capabilities of these tumor markers were found to be good (12). Since this initial subclassification, a fourth subclass was added as the classical group which was associated with EGFR mutations and a lack of TP53 mutations as well as mutations of the RB pathway. This subdivision was found to improve treatment and prognosis of patients with GBM of this specific genetic morphology (13).

While the traditional therapy for GBM resided in a surgical approach, the improved classification of GBM according to genetic morphology has increased the therapeutic capabilities of medicinal approach. The chemotherapy regimens focus exploitation of these tumor genetics. Patients found to have GBM with epigenetic silencing of MGMT DNA repair gene have significantly improved prognosis when treated with an alkylating agent such as temozolomide (TMZ) (14). When coupled with radiation (25 Gy), this therapy, known as Stupps regimen, is known to induce DNA double strand breaks and significantly improve patient prognosis. Additionally, it has been found that the addition of O-6-benzylguanine further enhances therapeutic effects of Stupps regimen (15). In addition to TMZ, carmustine and fotemustine have also been reported to have some therapeutic effect and are often used in combination with surgery. EGFR may also be targeted with bevacizumab which is a monoclonal antibody designed as an anti-angiogenic agent which has been found to significantly improve the prognosis of the classical subclass of GBM (16).

GBM is also associated with several cancer syndromes due to germline gene mutations including Li-Fraumeni syndrome (associated with P53), Neurofibromatosis 1/2 (NF-1 and NF-2 genes respectively), Turcots syndrome and BRCA syndrome. Additionally, these syndromes may be accelerated by predisposing events, however this has not been clearly established in the literature (17).

## 2 Case report

**Figure 1:**
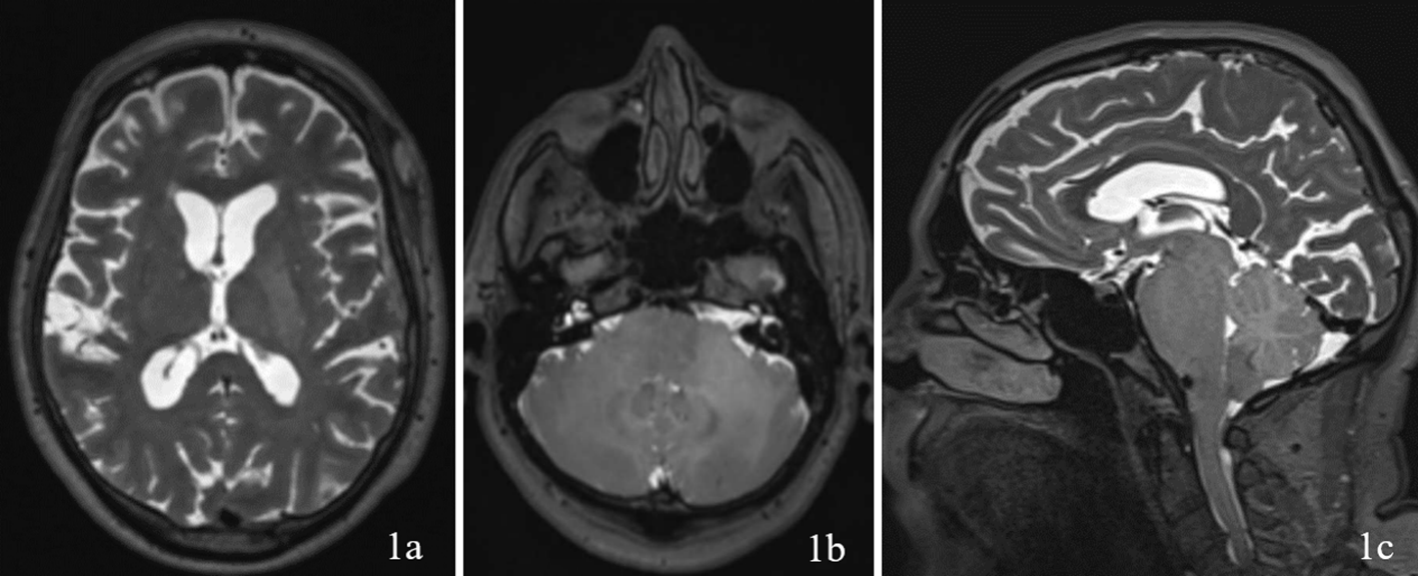
Imaging with T2WI sequence on 1a (axial through internal capsule/corona radiata), 1b (axial through pons/cerebellum), and 1c (sagittal) demonstrating diffuse hypodensity of the left internal capsule and left corona radiata.

**Figure 2:**
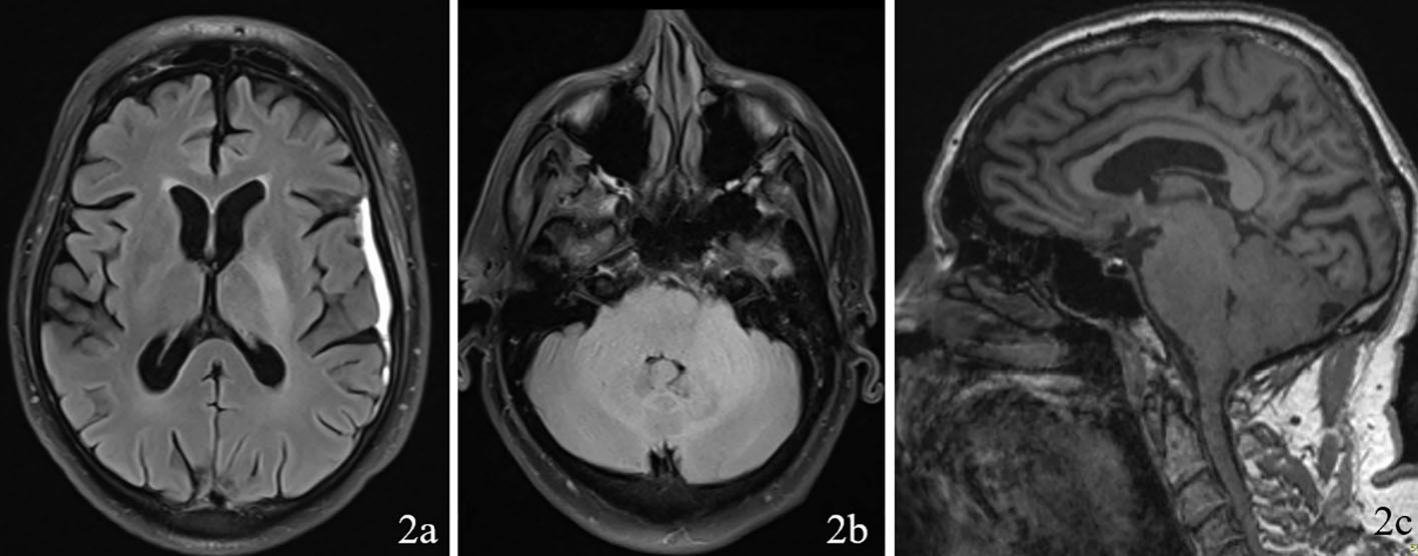
Imaging with FLAIR sequence on 2a (axial through internal capsule/corona radiata), 2b (axial through pons/cerebellum), and 2c (sagittal) demonstrating diffuse hypodensity of the left the pons and cerebellum.

A 69 year old male patient with a past medical history of hypertension, and right eye blindness for 11-12 years due to retinal vein thrombosis was referred to us for subdural hemorrhage. The patient had been taken to an outside hospital and had a CT taken showing a left temporal subdural hemorrhage (5mm x 7cm x 4 cm) and a lumbar puncture. The patient was then transferred to our ED and an additional CT was taken confirming the subdural hemorrhage, which was felt to be stable. The patient reported he had fallen and hit his head. The patient denied dizziness, chest palpitations, seizures or unilateral weakness and stated that he did not lose consciousness during the incident. Furthermore, the patient had similar events several times over the past 7 to 8 months along with symptoms of progressive worsening gait and headaches which he had been seen by outside institutions. Upon questioning the patient revealed a history of symptoms including difficulty ambulating, bilateral tongue and facial numbness and dysarthria. Subsequently, further imaging was completed and MRI revealed a diffuse hypodensity of the pons and cerebellum which had extension into the left internal capsule and left corona radiata. (**Figures 1**, **2**)

As no biopsy had been previously conducted, we felt that it was necessary to perform a biopsy for tissue diagnosis. A right sided retrosigmoid skull-base craniectomy approach for microsurgical right sided cerebellopontine angle approach was planned with the intention of conducting cerebellar and lateral pons biopsies. These biopsies were to be at the inferolateral aspect of the trigeminal nerve root entry zone.

The patient was brought into the operating room (OR) and was adequately prepped and positioned to exposed the right retrosigmoid area. The incision was planned with reference to the transverse sinus. The incision was made in a “C” fashion and then dissection revealed the suboccipital bone and mastoid. A single bur hole was placed and then enlarged to expose the inferior aspect of the transverse sinus and posterior portion of the sigmoid sinus. After this, we reached the signmoid transverse junction and via microscopy dissected the dura in a T-fashion. After encountering the cerebellum, the biopsy was planned with the Stealth navigation equipment after which we continued further to the pons and took an additional biopsy using the same technique. These were sent to pathology who reported that the cerebellum was in fact normal tissue; while the pons was nondiagnostic. Consequentially, additional permanent samples of the pons were taken at the inferolateral area of the trigeminal nerve root entry one. The patient was then closed appropriately and extubated without additional complications.

**Figure 3:**
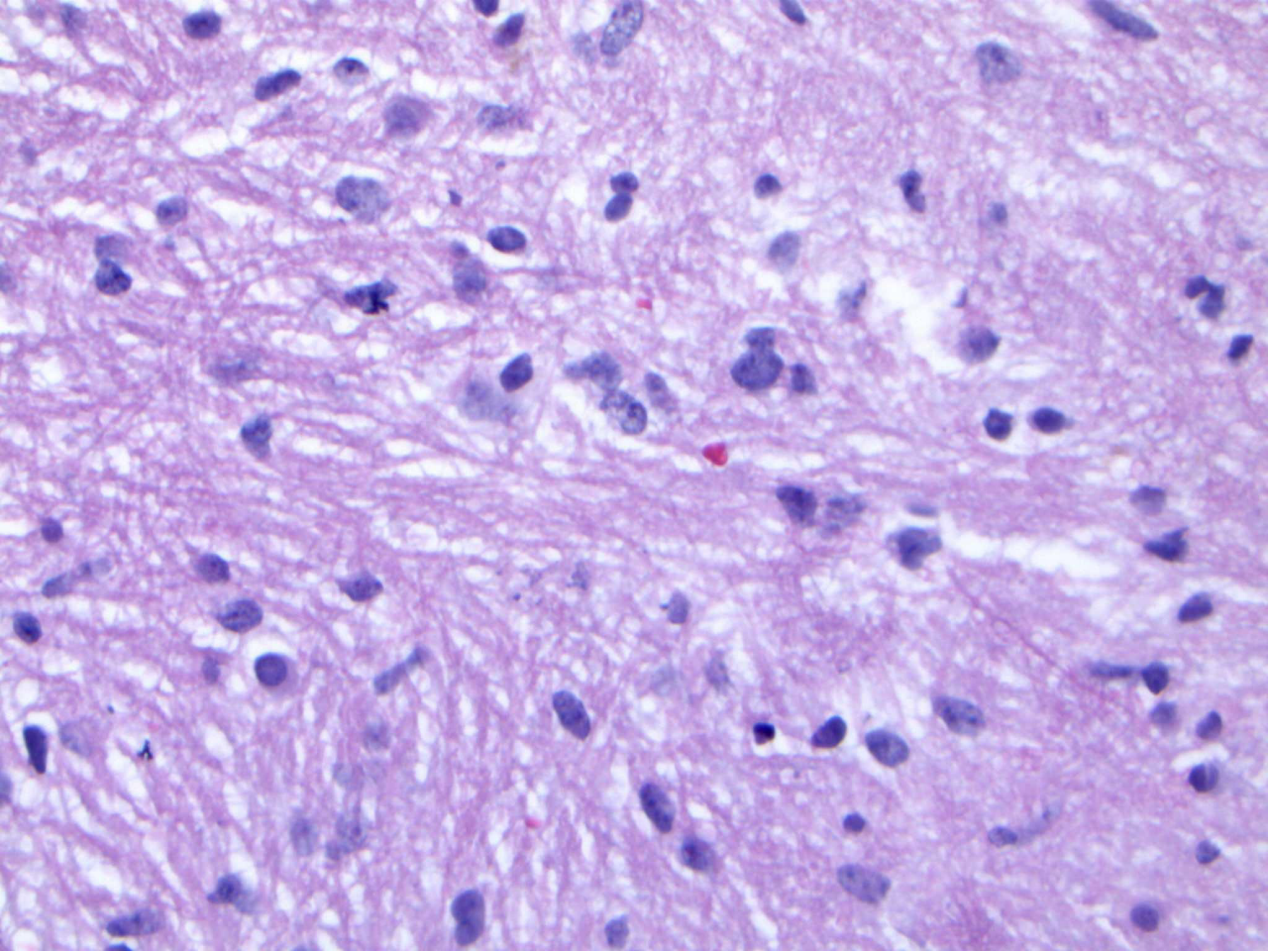
Hematoxylin and eosin (H&E) staining of tumor biopsy. Distinct features of atypical glial infiltrate are not definitively demonstrated and it is not feasible to establish a morphologic diagnosis of glioblastoma given the pontine location, as a biopsy sufficient to establish such a diagnosis would likely kill the patient.

**Figure 4:**
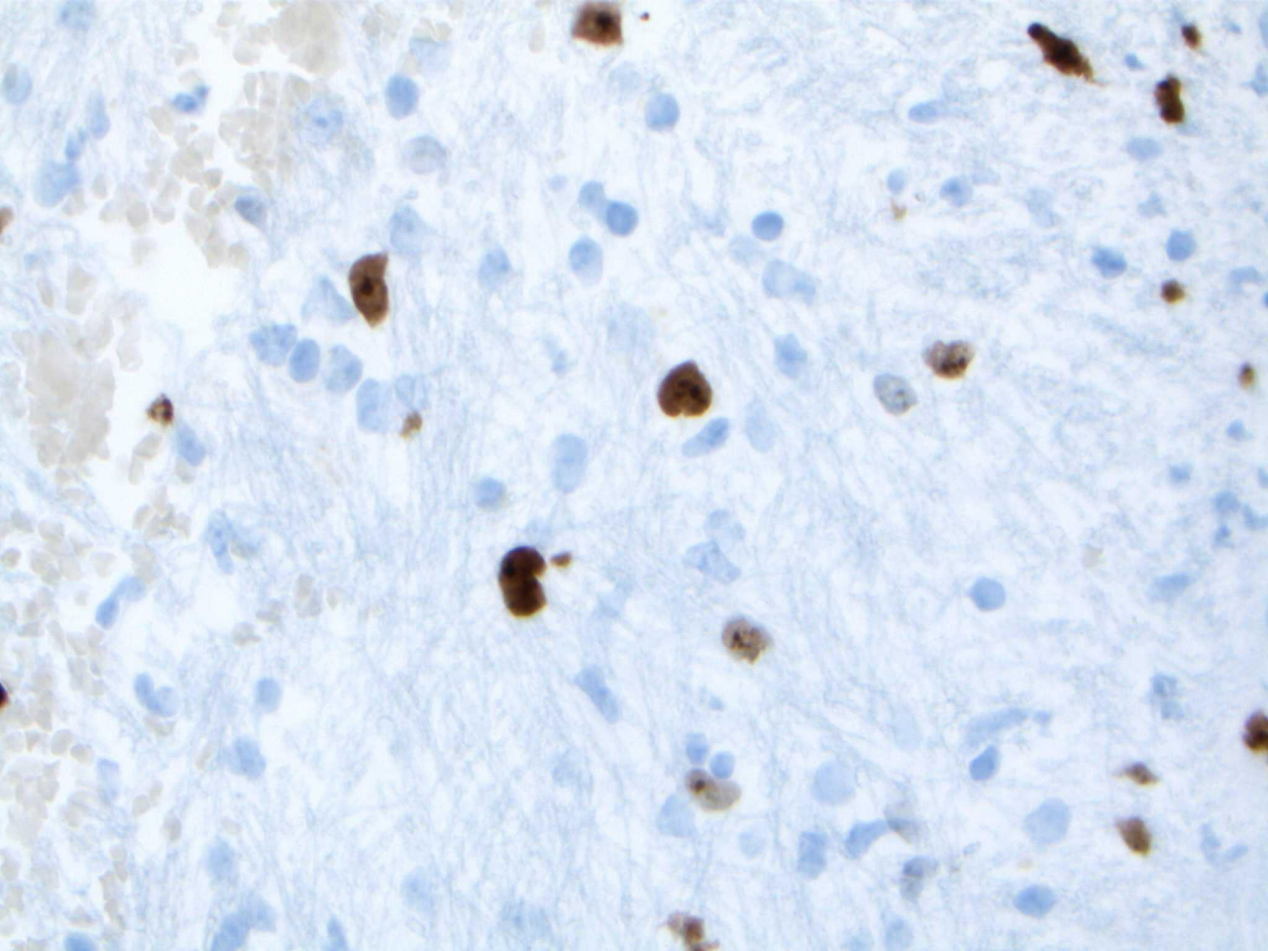
Nuclei labeling Ki-67 highlighting scattered atypical cells.

Upon reviewing the second set of permanent tissue samples, pathology identified a somewhat atypical glial infiltrate (**Figure 3**) with the majority of the astroglia nuclei labeling Ki-67 (**Figure 4**). Coupled with the radiology data (**Figures 1**, **2**), these results were only somewhat suggestive of an infiltrating astroglia neoplasm. To obtain enhanced characterization of the lesion, IDH1/R132H, ATRX, H3K27me3, and p53 immunohistochemical studies were conducted. However, these studies revealed no molecular signatures typical of astroglia neoplasia. Subsequentially, additional genetic studies were performed at Mayo Clinic Laboratory. These results indicated a mutant TERT gene promoter and PIK3R1 mutant as well as wild type for IDH1/2, ATRX, and TP53. These genetic results were suggestive as a glioblastoma WHO grade 4 tumor. Furthermore, the negative result for H3K27 was used to rule out the possibility of a diffuse midline glioma.

The final diagnosis of the patient according to molecular subtyping was a TERT promoter mutation which supported an integrated diagnosis of glioblastoma. Furthermore, the lack of IDH, ATRX, or TP53 mutations was suggestive of an aggressive glial neoplasm. The whole point of this challenging pathologic diagnosis is that it is primarily based on the molecular findings identified on NGS testing of the very limited biopsy sampling. This diagnosis thus falls under the category of “molecular glioblastoma”, and that it is not possible to establish a morphologic diagnosis of glioblastoma given the pontine location, as a biopsy sufficient to establish such a diagnosis would likely kill the patient. As such, figures providing histologic proof of glioblastoma identity do not exist.

In accordance with the molecular subtype of our patients GBM, we treated the patient with the traditional STUPP regimen (radiotherapy (4848.0 Gy administered in 21 fractions) plus concomitant temozolomide). After completion of this initial therapy, MRI revealed no new tumor progression. The patient was then maintained via STUPP protocol which was scheduled for twelve cycles of temozolomide (dosing 150 mg/m2) on a 28-day cycle. Due to side effects related to the chemotherapy treatment the patient requested a break from treatment at the sixth iteration. Thus, upon completing the fifth iteration, maintenance treatment was temporarily discontinued. However, upon follow-up MRI the patient demonstrated showed two areas of focal enhancement, one at the right middle cerebellar peduncle and the second at the left parietal subependymal region along the posterior aspect of the body of the left lateral ventricle. Maintenance therapy was reinitiated at this point and focal radiation was considered.

One month after re-initiation of treatment (13 months after initial start of chemotherapy), MRI demonstrated mild increase in size of the right cerebellar peduncle rim enhancing lesion (9mm x 6mm x 11mm as compared to 7mm x 5mm x 8mm). The lesion along the left parietal lobe subependymal region was stable at this point. At this point it was decided to administer focal proton therapy (30 Gy in 10 fractions) at the right cerebellar peduncle rim enhancing lesion along with continuation of maintenance temozolomide therapy.

At last follow-up the patient had demonstrated limited symptomatic improvement. Oral sensation has improved and the patient handling of oral secretions has improved. Although the patient still reports some numbness of the lower lip. Additionally, articulation of speech is much improved and coughing is reduced. Furthermore, the patient can maintain eye contact and demonstrates improved cognitive awareness.

## 3 Discussion

While supratentorial glioblastomas are among the most common type of glioblastomas, infratentorial glioblastomas are exceedingly rare tumors with an incidence rate of approximately 1.2% (18). In a recent multicenter retrospective study conducted by Weber et al., the median age of cerebellar GBM was reported to be 50.3 years, and 20% of the patients had brainstem invasion. The survival rate for these patients was found to be 14.7%, and brainstem invasion was identified as a poor prognostic factor (19). The clinical presentation of infratentorial GBM is similar to that of supratentorial GBM, with symptoms such as ataxia, dysmetria, tinnitus, dysarthria, and hemiparesis reported in both cases (18). However, in the presented case, the patient had no remarkable supratentorial spread of GBM contributing to the longstanding symptomatic progression prior to diagnosis (7-8 months). Reports of purely infratentorial GBM are rare. Magoha et al. described a homogenously hypointense ring enhancing lesion in the right brain stem in a young female patient presenting with right sided headache, hemiparesis, and tremor. Histologic examination was suggestive of GBM. Magoha et al. also reported temozolamide therapy with adjuvant radiotherapy was found to be useful in treating the malignancy (20). Additionally, Newton et al. described a 13 year old male with a pontine GBM which metastasized to the peritoneal cavity (which was attributed to a ventriculoperitoneal shunt) (21). Salas et al. reported an infratentorial GBM in a newborn which had a similar genetic profile to the tumor in this case report, mainly that it had no mutations in association with the TP53 gene (22). Lastly, Stark et al. reported only seven cases of infratentorial GBM out of 577 patients with GBM, with two patients presenting with GBM of the brainstem (18). Stark et al. concluded that the Ki67 and GFAP expression of supratentorial GBM bore no differences from infratentorial GBM. Lastly, Stark et al. also found temozolomide to be an effect therapeutic intervention for patients with infratentorial GBM (18).

Diagnosing infratentorial GBM can be challenging, as these tumors often have non-specific radiologic features (23). Of the patients presented by Stark et al., the first patient presented with symptoms of right hemiparesis, and the second patient had symptoms of vertigo, facial palsy, and dysphagia, with a survival time of 52 and 40 weeks, respectively (18). The second patient presented with left oculomotor palsy and hydrocephalus. These symptoms reminisce our patients’ symptoms. Furthermore, within infratentorial GBM in general the most frequently reported symptoms were ataxia, dysmetria, dysarthria, hemiparesis, and vertigo (18, 20–22).

It is hypothesized that infratentorial GBM may represent metastatic processes from supratentorial GBM dispersing through the cerebrospinal fluid (24). Our patient did not present with any imaging suggestive of a supratentorial GBM, suggesting that our patient presented with a primary GBM of the pons. The tumor described by Salas et al. was in a newborn which was used as evidence to support the claim that primary infratentorial GBM may represent a developmental pathology (22). However, the occurrence of the genetically similar primary infratentorial GBM in this case report occurring in an older man suggests that this process is not necessarily limited to developmental anomalies. Lastly, it is important to note that the two previously reported cases of brainstem GBM presented much younger than our patient and had shorter associated survival times.

## 4 Conclusion

In conclusion, we presented a case of a unique diffuse primary pontine glioblastoma multiforme, which was managed with radiotherapy plus concomitant and adjuvant temozolomide. Our treatment approach was based on the molecular subtype of the patient’s GBM, and was in accordance with the standard treatment for supratentorial GBMs. Despite aggressive treatment, the prognosis for patients with infratentorial glioblastoma multiforme remains poor. Further studies are needed to improve our understanding of the biology of this rare subtype of glioblastoma and develop more effective treatment strategies. Nevertheless, our case highlights the importance of personalized medicine and the use of molecular profiling to guide treatment decisions in patients with glioblastoma multiforme. We hope that this report will contribute to the growing body of knowledge on diffuse pontine glioblastoma multiforme and help clinicians make more informed treatment decisions for their patients.

## Data availability statement

The original contributions presented in the study are included in the article/supplementary material. Further inquiries can be directed to the corresponding author.

## Ethics statement

Written informed consent was obtained from the individual for the publication of any potentially identifiable images or data included in this article.

## Author contributions

BE: Writing – original draft, Writing – review & editing. VG: Writing – review & editing. ML-g: Writing – review & editing. JD: Writing – review & editing. MK: Writing – review & editing.
